# The impact of medical insurance on family financial support: evidence from CHARLS in China

**DOI:** 10.1186/s13561-023-00452-9

**Published:** 2023-07-20

**Authors:** Yuan Cao, Hua Chen, Xiaoxu Yang

**Affiliations:** 1 School of Finance, Chongqing Technology and Business University, Nan’an District, Chongqing, 400067 China; 2 School of Insurance, Central University of Finance and Economics, Changping District, Beijing, 102206 China

**Keywords:** Medical insurance, Family financial support, Out-of-pocket ratio of medical expenses, Health expense

## Abstract

**Background:**

In China, public medical insurance has expanded rapidly in the past 20 years. Many studies have discussed the benefits of medical insurance in improving residents’ health and financial stability, and increasing the utilization of medical services. Less attention is paid to the effect of medical insurance on family support between parents and children. This study focuses on the effect of medical insurance on promoting family financial support in China.

**Method:**

Fifty-five thousand sixty-two individual samples were obtained from four waves of the China Health and Retirement Longitudinal Study (CHARLS): 2011, 2013, 2015 and 2018. Linear-regression model and propensity score matching are used to determine the relationship between medical insurance and family financial support. Then, mediation model is introduced to identify the mediation mechanisms. Also, moderation model is used to estimate the moderation effect of parental education and health.

**Results:**

Medical insurance has significantly increased family financial support between the insured parents and their children. Moreover, this positive effect is heterogeneous since only families living in rural areas were affected, and the direction of family financial support changed with the aging of the parents. The welfare of medical insurance on financial status have also been proven in this paper. The results indicate that medical insurance reduces the out-of-pocket ratio of medical expenses and increases health investment, which can perform as as two mediation mechanisms to affect family financial support. Besides, the education and health status of the insured parents play a role in moderating the effect of medical insurance.

## Introduction

The public medical insurance system for urban workers was established in 1998, and since then, China’s medical insurance coverage has been expanded to include both urban and rural residents. By the end of 2022, the number of residents enrolled in Urban and Rural Resident Basic Medical Insurance (URRBMI) had reached 983 million, and the national coverage rate had increased rapidly from 64.6% in 2009 to more than 95%.^1^ A growing body of studies has shown that, as an important policy to transfer health risks and medical expenses, public medical insurance provides important health benefits and financial benefits to residents. Specifically, evidence from Critical Illness Insurance (CII) in rural China indicates that medical insurance has a positive effect on eliminating health inequalities and promoting the daily consumption of rural residents [1]. Evidence from New Rural Cooperative Medical Scheme (NCMS) shows that the enrollment in NCMS is associated with a lower incidence of catastrophic health expenditure [2] and better performance in activities of daily living and cognitive function [3]. Similarly, a study of expansions in public health insurance in the United States also indicates that an exposure to public health insurance in one’s childhood has positive effects on both economic and health outcomes in adulthood, such as reducing out-of-pocket medical spending, increasing financial stability, and decreasing the probability and mortality of chronic disease [4]. In addition, a comparative analysis of different medical insurances (i.e., NCMS, Urban Employee Medical Insurance (UEMI), and the Urban Resident Medical Insurance (URMI) of China) finds that different reimbursement benefits may induce inequity in health service utilization among middle-aged and older adults [5].

While most of the existing research on medical insurance has focused on its direct effect on health outcomes, medical utilization, and financial protection, the essential benefit of medical insurance to regulate family support and intergenerational relations has received little attention. Admittedly, the effect of medical insurance on private financial decision, such as family financial support within a family, is not as noticeable as its direct effect on improving health status and reducing medical expenses. However, it still deserves closer attention, considering that Chinese attach great importance to mutual support and material exchanges between their families. It is highly possible that the expansion of medical insurance in China can work as a public channel, which firstly influence the financial status of the insured and then influence their financial support between the children. As a result, the medical insurance can affect the net welfare of the family members.

Based on the preliminary assumption that the benefits from the public channel will affect the financial support from the private channel, this study empirically examines the impact of medical insurance on family financial support, based on panel data from the China Health and Retirement Longitudinal Study (CHARLS). China is a good case for this study because it has experienced rapid expansion in its medical insurance for urban and rural residents. What’s more, the Chinese attach great importance to family support between parents and children. Frequent and intensive family financial support between parents and children is considered an essential symbol of family harmony and family happiness.

This study contributes to the literature on medical insurance and family welfare in three ways. First, most previous studies on the effects of insurance programs on family support have focused on public pension programs, while the effects of medical insurance are less discussed. As a part of social security systems, URRBMI may also crowd out or crowd in family support, resulting in a change in the welfare of the families. Therefore, exploring the effect of medical insurance on family support is essential for evaluating the net welfare of medical insurance. Second, we consider the heterogeneous effects of medical insurance, which has received scant attention in previous studies about public support and private support. Third, we introduce two mechanisms by which medical insurance affects family financial support. Thus, we are able to provide more comprehensive explanations for the inconclusive answers regarding whether and how medical insurance affects family financial support.

The remainder of this study is organized as follows. We first introduce the background of the basic medical insurance of China. In the following two sections, we review the relevant literature and provide a theoretical analysis. Data, descriptive statistics, and empirical framework are subsequently presented. Then, we present the empirical results and provide a discussion on the results. In the last section, we give a conclusion of this paper.

## Background

In 1998, China established the UEMI as a supporting program for the reform of state-owned enterprises. The UEMI was the first formal medical insurance program in the country. Since then, a series of programs have been launched to provide medical services to both rural and urban residents. In 2003 and 2007, China established the NCMS and the URMI, respectively. The former aims to cover the vast majority of rural residents and the latter is designed to cover unemployed urban residents. Thus far, China has essentially achieved the goal of providing universal coverage of basic medical insurance for its citizens.

Since 2016, many regions have merged NCMS with URMI and implemented a unified URRBMI. Therefore, the coverage, financing mechanism, treatment, catalog, and management of NCMS and URMI have achieved equality between urban and rural residents. URRBMI employs a combination of individual contributions and financial subsidies financing mechanisms, residents can enjoy the benefits from URRBMI after paying the premiums for that year. What’s more, the financing standard and government subsidies of URRBMI have been continuously improved since its establishment. According to the requirement of the National Medical Security Bureau, the individual payment standard of URRBMI in 2022 is 350 yuan per capita, and the financial subsidy standard is no less than 610 yuan per capita. After enrollment, residents can enjoy the benefits from URRBMI for inpatient and outpatient medical expenses. For the inpatient medical expenses incurred in the first, second and third level hospitals, the payment line, i.e., the deductible of URRBMI is 200 yuan, 400 yuan, and 600 yuan respectively. The payment proportions for the expenses between the payment line and maximum payment line are 65%, 60%, and 50% respectively. As for the outpatient expenses, the payment line is 600 yuan. The payment proportion for the expenses between the payment line and the maximum payment line is 50%. The maximum annual payment is 3000 yuan per capita.

So far, a large number of literatures have studied the remarkable policy effects of URRBMI, including reducing medical burden [6] and the intensity of catastrophic health expenditure, ameliorating the impoverishment of the poorest residents [7], improving the benefit for outpatient care [8], and improving the health status of preschool children [9]. Several bodies of literature also estimate the impact of URRBMI on individual and family decisions, such as job selection [10] and stock market participation [11].

## Literature review

Many scholars have studied the impact of public policies on family financial support from both theoretical and empirical views, but most of them focused on public pension programs and ignored the potential effect of medical insurance. In the theoretical field, scholars generally believe that if a public program achieves its expected effect and improves the economic and health status of the insured, then both the insured parents and their children will change their financial support to each other. Whether a public program will crowd out or crowd in family financial support depends on the people’s motivation. For example, Becker and Barro point out that under altruistic motivation, the improvement of parents’ economic or health status will crowd out family financial support from their children [12, 13]. However, Cox believes that due to exchange motivation, children expect their parents to offer more help or inheritance to them after insurance enrollment. Thus, they increase the financial support to their insured parents in order to obtain these benefits in the future [14]. As a result, the improvement of parents’ economic or health status via insurance will crowd in family financial support from children. As for family financial support from parents to children, studies have shown that after economic or health status improves due to insurance coverage, parents tend to increase their financial support to children because of the behavioral habit of taking care of their children or in exchange for companionship and spiritual support [15, 16].

In the empirical studies, Deindl and Brandt study the social insurance systems of 14 European countries and find that social insurance will crowd out family support received by parents and the intensity of the crowd-out effect increases with the amount of social insurance benefits [17]. Lin et al. find that formal insurance has a crowd-out effect on informal private support through a theoretical model, and further verify this theory through experimental economics [18]. More specifically, scholars have proved that the Long-term Care Insurance (LTCI) in the United States, the public old-age support system in South Korea, and the New Rural Resident Pension policy in China have negative effects on family support from children to parents [19–21]. However, little is known about the influence of medical insurance programs on family support, especially for those financial support from parents to children. Deng et al. uses qualitative methods and finds that urban families, who tend to have more access to public welfare due to China’s dualistic welfare system, are less eager to invest in intergenerational financial support or expect less reciprocity. Thus, more empirical evidence from the medical insurance program needs to be provided [22].

Although medical insurance is less discussed as a public channel which may affect the private channel within the family, many studies have sufficiently confirmed its effect on reducing the medical expense, improving the health status and financial expectations, and stimulating the consumption demand of the insured [3, 23, 24]. In addition, studies that concentrate on the determinant of individual financial behavior have shown that a household’s financial literacy [25], financial education [26], financial status [27], number of children and the gender pattern of children [28] can affect the family support such as financial transfers. Therefore, the change in out-of-pocket medical expense and consumption demand may act as mediators to regulate the effect of medical insurance on family financial support. For example, when faced with less pressure from medical bills, insured parents may increase financial support and labor support to their children. Furthermore, children may increase the financial support to their insured parents to meet their increased demand for consumption.

Based on the above literature, we can conclude that the existing studies have conducted in-depth research on the effect of benefits from public channel on private support. However, these studies mainly focus on public pension programs and have yielded inconclusive results. Moreover, existing studies generally use cross-sectional data, and thus it is difficult to provide a convincing examination of the causal relationship between insurance and family support. In addition, the heterogeneous effect and mediation mechanism are seldomly addressed in the existing literature. To address the aforementioned problems, this study uses the CHARLS panel database to analyze the impact of URRBMI on family financial support and investigate the heterogeneity and mediation mechanism of its effect comprehensively.

## Theoretical analysis

When analyzing the effect of medical insurance on residents’ decision-making, existing literature often starts from two theoretical perspectives: Health performance and economic performance of medical insurance. Health performance refers to that medical insurance is expected to improve the health level of insured individuals. Economic performance refers to that medical insurance is expected to reduce medical expenses and improve the financial status of insured individuals. As an important component of the social security system, the health and economic performance of medical insurance have been well-discussed and demonstrated in previous studies. In terms of health performance, studies have indicated that basic medical insurance in China could boost preventive care utilization and increase citizens’ self-rated health [29–31]. In terms of economic performance, studies also found a significant effect of medical insurance in reducing citizens’ medical expenses and poverty rates [6, 32] and increasing nonmedical-related consumption and daily consumption [1, 23]. Therefore, theoretically speaking, if the URRBMI achieve the expected results and improve the health and economic status of insured individuals, it is highly possible to further affect their financial decisions, such as the transfers between themselves and their children.

As for the effect of medical insurance on financial support from insured parents to children, scholars generally believe that with the improvement of economic and health conditions, the dependence of elderly people on their children can be weakened, and they tend to have more ability to provide financial assistance to their children, i.e., increase their financial support to children. This effect can be more significant in China because Chinese parents attach higher importance to family support and have stronger behavioral inertia in supporting children [15]. Moreover, Chinese parents may also increase their financial support to children in exchange for children’s spiritual support, such as companionship and household care.

As for the effect of medical insurance on financial support from children to insured parents, studies consider that it depends on what kind of motivation the children hold. Generally, there are two kinds of motivation that can produce opposite results: Altruistic Motivation and Exchange motivation. Under “Altruistic Motivation”, children tend to reduce financial support for elderly parents if the health and economic conditions of their parents can be improved by medical insurance, which means that medical insurance crowd out financial support from children to parents. The portion of the financial support that was originally transferred to parents will be returned to children, thereby maximizing the utility of the offspring generation [12, 13]. While under “Exchange motivation”, the improvement of parents’ health and economic conditions by the medical insurance might increase the labor support, financial support, and inheritance that parents can provide to their children. In order to obtain more support or inheritance from the parents, children will increase their financial support to the elderly parents, which means that the medical insurance crowed in financial support from children to parents [14].

## Data and descriptive statistics

### Data and variables

Our data comes from the CHARLS database in 2011, 2013, 2015, and 2018. CHARLS uses multistage stratified probability proportional to size sampling to conduct a nationwide survey of respondents aged 45 years or older. The survey follows detailed protocols for sampling, field surveying, and data quality verification. The baseline CHARLS was conducted in 2011. The second-, third-, and fourth-wave national surveys conducted in 2013, 2015, and 2018 aimed to revisit the same respondents sampled in the first wave. The information in CHARLS includes individual demographic backgrounds, family contacts and transfers, health care, and insurance. Thus, this dataset has been widely used in international health economics research, aging research, and family welfare analysis [33–35].

By the time the national follow-up was completed in 2018, the survey had covered 150 county-level units and 450 village-level units in 28 provinces, with 19,000 yearly respondents in approximately 12,400 families. We generate a series of variables required in our study based on the original database and questionnaires, such as URRBMI, family financial support from parents to children, and family financial support from children to parents. To identify the impact of URRBMI on family financial support, we exclude respondents who were enrolled in other social medical insurance, such as UEMI and Supplementary medical insurance. The participants from commercial medical insurance are also excluded from the full sample. Since NCMS and URMI in some areas were merged into URRBMI after 2016, both those enrolled in NCMS and URMI are regarded as being enrolled in URRBMI in this study. Specifically, those enrolled in NCMS, URMI, and URRBMI constitute the treatment group. After excluding respondents with missing information, we finally obtain unbalanced cross-sectional data on 55,062 observations. We define the treatment group as “the parents enrolled in URRBMI” and the control group as “the parents without any medical insurance.” There are 52,734 samples in the treatment group and 2,328 samples in the control group.

The independent variable in our study is URRBMI, the value of insured is 1, and the value of non-insured is 0. The dependent variables are ${Support}_{p-c}$ and ${Support}_{c-p}$, which capture family financial support from parents to children and family financial support from children to parents, respectively. We aggregate the money support in total (includes providing living expenses, paying for water, electricity or telephone bill, paying for mortgage/ rent or other forms of regular expenses in the past year) and the total value of in-kind payment support (such as buying food, clothes or other items in the past year) as the total family financial support a parent has received from his/her children. The same strategy is also used to measure the family financial support from parents to children.

We are also interested in two factors that may mediate the effect of URRBMI on family financial support: the out-of-pocket ratio of medical expenses and health expense. The out-of-pocket ratio of medical expenses is equal to out-of-pocket inpatient medical expenses in the past year divided by the total inpatient medical expenses in the last year. Health expense is equal to the expense of health investment and health care in the last year. The control variables in our analysis include both individual-level and household-level characteristics. The individual-level variables include age, gender, urban residence, marriage status, education, self-rated health status, and public pensions. The household-level variables include the number of children, living arrangements, and family financial assets.

### Descriptive statistics

Table 1 shows the definition and descriptive statistics of the variables. We compare the differences between the treatment group and the control group with the t-test and find that the two groups show significant differences in family financial support. The average of family financial support from URRBMI-insured parents to children is 4,196 yuan per mouth, which is significantly higher than that of uninsured parents (2,925 yuan per month). This significant difference in family financial support from parents to children initially indicates that URRBMI may have a positive effect on parental financial support to children. Similarly, the average of family financial support from children to URRBMI-insured parents (3,882 yuan per month) is significantly higher than that of those uninsured by URRBMI (2,421 yuan per month), which may be due to exchange motivation from children. In addition, compared with uninsured respondents, insured respondents are younger, better educated, more likely to be married, and have more family financial assets. On the other hand, uninsured respondents are healthier, have lower public pension enrollment rates, and are more likely to have more children and live with children. The characteristics of the uninsured group suggest that they may be more dependent on family and children for financial support.

**Table 1:** Descriptive statistics

**Variables**	**Definition**	**Treatment group**	**Control group**	**Differences**
**Independent variable**				
URRBMI	1 if the respondent participates in URRBMI; 0 if not			
**Dependent variables**				
Support_p-c_	Total family financial support from the respondent to his/her children	4196.393	2925.427	1270.996***
Support_c-p_	Total family financial support from children to the respondent	3882.124	2420.817	1461.307***
**Control variables**				
Age	Age of the respondent	60.664	61.509	-0.845***
Male	1 if the respondent is male; 0 if female	0.48	0.428	0.052***
Urban residence	1 if the respondent is urban resident; 0 if rural resident	0.203	0.312	-0.108***
Marriage status	1 if the respondent is married or cohabitating; 0 if separated, divorced or never married	0.883	0.806	0.077***
Education:				
No formal education	1 if the respondent has no formal education; 0 if not	0.245	0.302	-0.057***
Can read and write	1 if the respondent’s highest level of education is “can read and write”; 0 if not	0.197	0.222	-0.024***
Primary school	1 if the respondent’s highest level of education is primary school; 0 if not	0.226	0.200	0.027***
Secondary school or above	1 if the respondent’s highest level of education is secondary school or above; 0 if not	0.332	0.277	0.055***
Self-rated health status	1 if very poor; 2 if poor; 3 if fair; 4 if good; 5 if very good	3.042	3.083	-0.042**
Public pensions	1 if the respondent participates in a public pension program; 0 if not	0.602	0.294	0.308***
Number of children	Total number of living and dead children, including biological children, stepchildren, and adopted children	3.029	3.162	-0.132***
Living arrangement	1 if the respondent living with his/her children; 0 if not	0.279	0.349	-0.069***
Family financial assets	Total net family financial assets	4708.932	3091.608	1617.324***

*, **, and *** indicate that the coefficients significantly differ from 0 at the 10%, 5%, and 1% levels, respectively

## Empirical framework

### Basic empirical model

In empirical analysis, the dependent variables ${Support}_{p-c}$ and ${Support}_{c-p}$ are individually scaled by their logarithmic forms. Specifically, we estimate the following linear regression model:

1 $${\varvec{L}}{\varvec{n}}({{\varvec{S}}{\varvec{u}}{\varvec{p}}{\varvec{p}}{\varvec{o}}{\varvec{r}}{\varvec{t}}}_{{\varvec{i}}{\varvec{t}}})={{\varvec{\beta}}}_{0}+{{\varvec{\beta}}}_{1}{{\varvec{U}}{\varvec{R}}{\varvec{R}}{\varvec{B}}{\varvec{M}}{\varvec{I}}}_{{\varvec{i}}{\varvec{t}}}+{{\varvec{\beta}}}_{{\varvec{X}}}{{\varvec{X}}}_{{\varvec{i}}{\varvec{t}}}+{{\varvec{\beta}}}_{2}{{\varvec{Y}}{\varvec{e}}{\varvec{a}}{\varvec{r}}}_{{\varvec{t}}}+{{\varvec{\beta}}}_{3}{{\varvec{P}}{\varvec{r}}{\varvec{o}}{\varvec{v}}{\varvec{i}}{\varvec{n}}{\varvec{c}}{\varvec{e}}}_{{\varvec{i}}}+{{\varvec{u}}}_{{\varvec{i}}{\varvec{t}}}$$

where $URRBMI$ is a binary variable, indicating whether the respondent is enrolled in URRBMI. ${\beta }_{1}$ is the estimator that we are most interested in since it captures the effect of URRBMI on family financial support. ${X}_{it}$ is a vector of individual-level and family-level control variables. ${Year}_{t}$ is a series of year dummy variables with the coefficient ${\beta }_{2}$ representing the year fixed effects. ${\beta }_{3}$ represents the province fixed effects and ${u}_{it}$ is the error term.

The linear regression model in Eq. (1) examines the effect of URRBMI by directly comparing the family financial support between the treatment and the control groups. However, it should be noted that enrolling in URRBMI is voluntary. Residents with higher income or greater risk awareness tend to have higher enrollment rates in insurance programs, and these wealthier respondents may also offer more financial support to their children. The unobserved heterogeneity between insured and uninsured individuals may lead to selection bias. The key point to solve this problem is to identify whether the family financial support for residents with URRBMI are higher than the potential results if they were not enrolled in URRBMI. In other words, it is necessary to determine whether there is a significant average treatment effect on the treated (ATT). In the robustness test, we use the propensity score matching (PSM) method to eliminate the influence of selection bias and estimate the ATT. Besides, another problem that may interfere with our estimation results is reverse causation. Since the data used in this study come from a microsurvey, the timing of URRBMI enrollment cannot be precisely identified. The enrollment time may not necessarily precede the occurrence of the family financial support, which may result in the endogenous problem of reverse causation. Therefore, we construct a new independent variable ${URRBMI}_{coverage}$ which captures the percentage of time that an individual was covered by URRBMI from 2011 to 2018. Using ${URRBMI}_{coverage}$ as the independent variable and family financial support in 2018 as the dependent variable, we can rule out potential reverse causality issues.

### Propensity score matching (PSM)

Since enrollment in URRBMI is not randomly assigned, the decision to enroll in URRBMI is likely to be affected by individuals’ personal characteristics. For example, people with higher education and stronger risk awareness are more inclined to enroll in medical insurance. Therefore, the observed differences in family financial support between the treatment group and the control group may be caused by two things: one is the effect of enrollment in URRBMI, and the other is the natural differences of the two groups. To eliminate the endogeneity problem caused by sample selection bias, we use the PSM method to check the robustness of the main results presented in the previous section. For individual $i$, family financial support may have two states, depending on whether or not $i$ is enrolled in URRBMI:

2 $${{\varvec{S}}{\varvec{u}}{\varvec{p}}{\varvec{p}}{\varvec{o}}{\varvec{r}}{\varvec{t}}}_{{\varvec{i}}{\varvec{t}}}\left\{\begin{array}{c}{{\varvec{S}}{\varvec{u}}{\varvec{p}}{\varvec{p}}{\varvec{o}}{\varvec{r}}{\varvec{t}}}_{1{\varvec{i}}{\varvec{t}}} \,\,\,\,if\, {{\varvec{U}}{\varvec{R}}{\varvec{R}}{\varvec{B}}{\varvec{M}}{\varvec{I}}}_{{\varvec{i}}{\varvec{t}}}=1\\ {{\varvec{S}}{\varvec{u}}{\varvec{p}}{\varvec{p}}{\varvec{o}}{\varvec{r}}{\varvec{t}}}_{0{\varvec{i}}{\varvec{t}}} \,\,\,\,if\, {{\varvec{U}}{\varvec{R}}{\varvec{R}}{\varvec{B}}{\varvec{M}}{\varvec{I}}}_{{\varvec{i}}{\varvec{t}}}=0\end{array}\right.$$

where ${Support}_{0it}$ represents the family financial support when individual $i$ is not insured, and ${Support}_{1it}$ represents the family financial support when individual $i$ is enrolled in URRBMI. Thus, Eq. (2) can be rewritten as:

3 $${{\varvec{S}}{\varvec{u}}{\varvec{p}}{\varvec{p}}{\varvec{o}}{\varvec{r}}{\varvec{t}}}_{{\varvec{i}}{\varvec{t}}}=(1-{{\varvec{U}}{\varvec{R}}{\varvec{R}}{\varvec{B}}{\varvec{M}}{\varvec{I}}}_{{\varvec{i}}{\varvec{t}}}){{\varvec{S}}{\varvec{u}}{\varvec{p}}{\varvec{p}}{\varvec{o}}{\varvec{r}}{\varvec{t}}}_{0{\varvec{i}}{\varvec{t}}}+{{\varvec{U}}{\varvec{R}}{\varvec{R}}{\varvec{B}}{\varvec{M}}{\varvec{I}}}_{{\varvec{i}}{\varvec{t}}}{{\varvec{S}}{\varvec{u}}{\varvec{p}}{\varvec{p}}{\varvec{o}}{\varvec{r}}{\varvec{t}}}_{1{\varvec{i}}{\varvec{t}}}={{\varvec{S}}{\varvec{u}}{\varvec{p}}{\varvec{p}}{\varvec{o}}{\varvec{r}}{\varvec{t}}}_{0{\varvec{i}}{\varvec{t}}}+\boldsymbol{ }({{\varvec{S}}{\varvec{u}}{\varvec{p}}{\varvec{p}}{\varvec{o}}{\varvec{r}}{\varvec{t}}}_{1{\varvec{i}}{\varvec{t}}}-{{\varvec{S}}{\varvec{u}}{\varvec{p}}{\varvec{p}}{\varvec{o}}{\varvec{r}}{\varvec{t}}}_{0{\varvec{i}}{\varvec{t}}}){{\varvec{U}}{\varvec{R}}{\varvec{R}}{\varvec{B}}{\varvec{M}}{\varvec{I}}}_{{\varvec{i}}{\varvec{t}}}$$

where ${(Support}_{1it}-{Support}_{0it})$ is the treatment effect of the URRBMI on family financial support. Since ${(Support}_{1it}-{Support}_{0it})$ is a random variable, we only focus on the expected value of the URRBMI treatment group, i.e., the ATT:

4 $${\varvec{A}}{\varvec{T}}{\varvec{T}}\equiv {\varvec{E}}{({\varvec{S}}{\varvec{u}}{\varvec{p}}{\varvec{p}}{\varvec{o}}{\varvec{r}}{\varvec{t}}}_{1{\varvec{i}}{\varvec{t}}}-{{\varvec{S}}{\varvec{u}}{\varvec{p}}{\varvec{p}}{\varvec{o}}{\varvec{r}}{\varvec{t}}}_{0{\varvec{i}}{\varvec{t}}}|{{\varvec{U}}{\varvec{R}}{\varvec{R}}{\varvec{B}}{\varvec{M}}{\varvec{I}}}_{{\varvec{i}}{\varvec{t}}}=1)$$

For individual $i$ of the treatment group, if individual $j$ can be found in the control so that the distance between ${X}_{i}$ and ${X}_{j}$ is as small as possible, i.e., ${X}_{i}\approx {X}_{j}$. Based on the ignorability assumption, the probability of individual $i$ and individual $j$ being enrolled in URRBMI is similar. Thus, ${Support}_{jt}$ can be taken as an estimator of ${Support}_{0it}$, i.e., $\widehat{{Support}_{0it}}={Support}_{jt}$. We can use $({Support}_{it}-\widehat{{Support}_{0it}})=({Support}_{it}-{Support}_{jt})$ as a measurement of the treatment effect on individual $i$.

We use the “propensity score” proposed by Rosenbaum and Rubin to measure the distance between ${X}_{i}$ and ${X}_{j}$ [36]. The propensity score of individual $i$ is the conditional probability of individual $i$ being enrolled in URRBMI given ${X}_{i}$, i.e., $P({X}_{i})\equiv P({URRBMI}_{it}=1|X={X}_{i})$. We use the logit regression to estimate the propensity score $P({X}_{i})$, and use the propensity score as a distance function for matching. The ATT can be estimated from

5 $${\varvec{A}}{\varvec{T}}{\varvec{T}}=\frac{1}{{{\varvec{N}}}_{{\varvec{t}}}}{\sum }_{{\varvec{i}}:{{\varvec{U}}{\varvec{R}}{\varvec{R}}{\varvec{B}}{\varvec{M}}{\varvec{I}}}_{{\varvec{i}}}=1}({{\varvec{S}}{\varvec{u}}{\varvec{p}}{\varvec{p}}{\varvec{o}}{\varvec{r}}{\varvec{t}}}_{{\varvec{i}}{\varvec{t}}}-\widehat{{{\varvec{S}}{\varvec{u}}{\varvec{p}}{\varvec{p}}{\varvec{o}}{\varvec{r}}{\varvec{t}}}_{0{\varvec{i}}{\varvec{t}}}})$$

where ${N}_{t}={\sum }_{i}{I}_{i}$ represents the number of individuals in the treatment group, and ${\sum }_{i:{URRBMI}_{i}=1}$ represents aggregating the treatment effect of individuals in the treatment group.

### Mediation model

If the independent variable ${URRBMI}_{it}$ influences the dependent variable *Support* by affecting some mediator variables, then we can use the following regression equation to describe the causal relationship between the variables. This framework for mediation analysis was first proposed by Baron and Kenny, based on the linear structural equation model [37]. It was further developed by Imai et al. and has been used by many social science practitioners to examine the mediation mechanism [38]. The significant effect of medical insurance on reducing medical expenses and releasing health investment has been well documented in previous research. These two factors are important components of family finance, and may further influence other family decisions, such as family financial support within the family. Therefore, the mediator mechanisms we focus on are the out-of-pocket ratio of medical expenses and health expense. First, medical insurance may reduce the out-of-pocket ratio of medical expenses, thus promoting family financial support from insured parents to children. Second, medical insurance may stimulate the health investment of insured parents, thus increasing the family financial support from children to insured parents.

6 $${{\varvec{M}}{\varvec{e}}{\varvec{d}}{\varvec{i}}{\varvec{a}}{\varvec{t}}{\varvec{o}}{\varvec{r}}}_{{\varvec{i}}{\varvec{t}}}={{\varvec{\beta}}}_{10}+{\varvec{a}}\times {{\varvec{U}}{\varvec{R}}{\varvec{R}}{\varvec{B}}{\varvec{M}}{\varvec{I}}}_{{\varvec{i}}{\varvec{t}}}+{{\varvec{\beta}}}_{1{\varvec{X}}}{{\varvec{X}}}_{{\varvec{i}}{\varvec{t}}}+{{\varvec{\beta}}}_{12}{{\varvec{Y}}{\varvec{e}}{\varvec{a}}{\varvec{r}}}_{{\varvec{t}}}+{{\varvec{\beta}}}_{13}{{\varvec{P}}{\varvec{r}}{\varvec{o}}{\varvec{v}}{\varvec{i}}{\varvec{n}}{\varvec{c}}{\varvec{e}}}_{{\varvec{i}}}+{{\varvec{u}}}_{{\varvec{i}}{\varvec{t}}1}$$

7 $${{\varvec{L}}{\varvec{n}}({\varvec{S}}{\varvec{u}}{\varvec{p}}{\varvec{p}}{\varvec{o}}{\varvec{r}}{\varvec{t}}}_{{\varvec{i}}{\varvec{t}}})={{\varvec{\beta}}}_{20}+{\varvec{c}}\times {{\varvec{U}}{\varvec{R}}{\varvec{R}}{\varvec{B}}{\varvec{M}}{\varvec{I}}}_{{\varvec{i}}{\varvec{t}}}+{{\varvec{\beta}}}_{2{\varvec{X}}}{{\varvec{X}}}_{{\varvec{i}}{\varvec{t}}}+{{\varvec{\beta}}}_{22}{{\varvec{Y}}{\varvec{e}}{\varvec{a}}{\varvec{r}}}_{{\varvec{t}}}+{{\varvec{\beta}}}_{23}{{\varvec{P}}{\varvec{r}}{\varvec{o}}{\varvec{v}}{\varvec{i}}{\varvec{n}}{\varvec{c}}{\varvec{e}}}_{{\varvec{i}}}+{{\varvec{u}}}_{{\varvec{i}}{\varvec{t}}2}$$

8 $${{\varvec{L}}{\varvec{n}}({\varvec{S}}{\varvec{u}}{\varvec{p}}{\varvec{p}}{\varvec{o}}{\varvec{r}}{\varvec{t}}}_{{\varvec{i}}{\varvec{t}}})={{\varvec{\beta}}}_{30}+{\varvec{c}}\boldsymbol{^{\prime}}\times {{\varvec{U}}{\varvec{R}}{\varvec{R}}{\varvec{B}}{\varvec{M}}{\varvec{I}}}_{{\varvec{i}}{\varvec{t}}}+{\varvec{b}}\times {{\varvec{M}}{\varvec{e}}{\varvec{d}}{\varvec{i}}{\varvec{a}}{\varvec{t}}{\varvec{o}}{\varvec{r}}}_{{\varvec{i}}{\varvec{t}}}+{{\varvec{\beta}}}_{3{\varvec{X}}}{{\varvec{X}}}_{{\varvec{i}}{\varvec{t}}}+{{\varvec{\beta}}}_{32}{{\varvec{Y}}{\varvec{e}}{\varvec{a}}{\varvec{r}}}_{{\varvec{t}}}+\boldsymbol{ }{{\varvec{\beta}}}_{33}{{\varvec{P}}{\varvec{r}}{\varvec{o}}{\varvec{v}}{\varvec{i}}{\varvec{n}}{\varvec{c}}{\varvec{e}}}_{{\varvec{i}}}+{{\varvec{u}}}_{{\varvec{i}}{\varvec{t}}3}$$

Specifically, the mediator variables in this study are ${Medical\, expenses}_{Out-of-pocket ratio}$ and $Health\, expense$. The coefficient $a$ in Eq. (6) represents the effect of ${URRBMI}_{it}$ on ${Mediator}_{it}$. The coefficient $b$ in Eq. (8) represents the effect of ${Mediator}_{it}$ on ${Support}_{it}$. The coefficient $c$ in Eq. (7) represents the total effect of ${URRBMI}_{it}$ on ${Support}_{it}$, and the coefficient $c{\prime}$ represents the direct effect of ${URRBMI}_{it}$ on ${Support}_{it}$ after controlling the effect of ${Mediator}_{it}$.

Baron and Kenny suggest that mediation effects can be tested under the following conditions [39]: First, the variation in ${URRBMI}_{it}$ is a significant predictor of the variation in ${Mediator}_{it}$ in Eq. (6); second, the variation ${URRBMI}_{it}$ is a significant predictor of the variation in ${Support}_{it}$ in Eq. (7); and third, the variation in ${Mediator}_{it}$ is a significant predictor of the variation in ${Support}_{it}$ in Eq. (8). The tested mediator is a valid mediator when all of these conditions are satisfied in the predicted direction, in which case the effect of ${URRBMI}_{it}$ on ${Support}_{it}$ must be smaller in Eq. (8) than in Eq. (7).

We also utilize Sobel–Goodman mediation tests to verify the robustness of our mediation analysis and estimate the ratios of the total effect that is mediated [40]. The Sobel–Goodman method directly tests the significance of the product of coefficient *a* and coefficient *b* in Eqs. (6) to (8), thus its testing power is superior to the sequential test, i.e., Baron and Kenny’s step-by-step method.

### Moderation model

The causal effect of URRBMI on family financial support might be affected by personal backgrounds, such as education level and health status. With higher education and better health status, parents may engage in more activities and generate more entertainment expenses, which may have a moderate effect on the financial support between themselves and their children. In addition, healthier parents may also have less medical and health care consumption, thus moderating the support between themselves and their children. Therefore, we use hierarchical regression to perform moderation analysis in this section. First, we repeat the regression between URRBMI and family financial support in Eq. (1). Other covariate variables, time fixed effect, and province fixed effect are also included. Second, education and self-rated health status are entered into Eq. (1) as independent variables. Third, the interaction terms of education and URRBMI, and self-rated health status and URRBMI are entered into Eq. (1) to examine the moderation effects.

## Empirical results

### The linear regression results

Firstly, we use ordinary least squares (OLS) regression to analyze the effect of URRBMI on family financial support. Table 2 reports the OLS regression results. The estimators in Column (1) and Column (2) present the effect of URRBMI on family financial support from parents to children without and with covariates, respectively. The results indicate that URRBMI has a positive and significant effect on family financial support from parents to children. Specifically, the total amount of financial support from parents to children increases by 21.9% for URRBMI enrollees, as shown in Column (1). This positive effect of URRBMI on financial support from parents to children remains significant after the addition of control variables, as shown in Column (2), with an increase of 18.7% for URRBMI enrollees. Since the average family financial support from parents to children of all samples is 4,143 yuan, this increase is equal to 775 yuan per year. The estimators in Column (3) and Column (4) present the effect of URRBMI on family financial support from children to parents without and with covariates, respectively. These results also indicate that URRBMI has a positive and significant effect on family financial support from children to parents. As shown in Column (4), the total amount of financial support from children to parents increases by 51.9% for URRBMI enrollees. Since the average family financial support from children to parents of all samples is 3,820 yuan, this increase is equal to 1,983 yuan per year.

**Table 2:** The effects of URRBMI on family financial support: OLS estimates

	**Ln (Support**_**p-c**_**)**	**Ln (Support**_**p-c**_**)**	**Ln (Support**_**c-p**_**)**	**Ln (Support**_**c-p**_**)**
	(1)	(2)	(3)	(4)
URRBMI	0.219*** (0.071)	0.187*** (0.070)	0.704*** (0.079)	0.519*** (0.074)
Age		-0.068*** (0.002)		0.066*** (0.002)
Male		-0.013 (0.033)		-0.231*** (0.032)
Urban residence		0.820*** (0.049)		-0.547*** (0.045)
marriage status		0.424*** (0.044)		0.350*** (0.045)
Can read and write		0.197*** (0.044)		0.143*** (0.043)
Primary school		0.364*** (0.045)		0.148*** (0.044)
Secondary school or above		0.673*** (0.048)		0.123*** (0.046)
Self-rated health status		0.127*** (0.016)		0.033** (0.015)
Public pensions		-0.161*** (0.036)		0.289*** (0.035)
Number of children		0.125*** (0.011)		0.279*** (0.011)
Living arrangement		-0.416*** (0.035)		-1.676*** (0.035)
Family financial assets		0.577*** (0.070)		0.148** (0.059)
Constant	0.780** (0.342)	2.996*** (0.373)	0.213 (0.324)	-3.682*** (0.346)
Observations	55,062	55,062	55,062	55,062
*R*-squared	0.102	0.151	0.095	0.216
Province fixed effects	Yes	Yes	Yes	Yes
Year fixed effects	Yes	Yes	Yes	Yes

*, **, and *** indicate that the coefficients significantly differ from 0 at the 10%, 5%, and 1% levels. The dependent variables are scaled by it’s logarithmic form. Family financial assets are scaled by 1/100,000

Additionally, the coefficient of respondents’ age on family financial support from their children to themselves is significantly positive, while the coefficient of respondents’ age on family financial support from themselves to their children is significantly negative. This indicates that the older an individual is, the more financial support he/she receives from his/her children, and the less financial support he/she gives to his/her children. The coefficient of urban residence indicates that rural residents tend to receive more financial support from their children, while urban residents tend to provide more financial support to their children. The above results preliminarily show that the elderly and rural parents are more dependent on family financial support, which may lead to the heterogeneity of the effect of URRBMI.

### Heterogeneity analysis

The results of heterogeneity analysis are shown in Table 3. The results in Columns (1) and (2) in Table 3 show that the positive effect of URRBMI on family financial support from parents to children and from children to parents for rural residents is significantly greater than that for urban residents. In Panel A, the total amount of family financial support from parents to children increases significantly by 14.4% for rural residents enrolled in URRBMI. In comparison, the total amount of family financial support from parents to children for urban residents increases by only 10.4%, and this effect is nonsignificant. Similarly, the results in Columns (1) and (2) of Panel B in Table 3 indicate the total amount of family financial support from children to parents increases significantly by 67.8% for rural residents enrolled in URRBMI, while this effect is not nonsignificant for urban residents.

**Table 3:** Heterogeneous effects of URRBMI on family financial support

	**Urban and rural residence**		**Age**	
	**Rural**	**Urban**	**Middle-aged**	**Elderly**
	(1)	(2)	(3)	(4)
**Dependent variables:** Ln (Support_p-c_)				
URRBMI	0.144* (0.079)	0.104 (0.144)	0.522*** (0.115)	0.482*** (0.096)
Observations	43,611	11,451	26,361	28,701
*R*-squared	0.147	0.145	0.203	0.166
Covariates	Yes	Yes	Yes	Yes
Province fixed effects	Yes	Yes	Yes	Yes
Year fixed effects	Yes	Yes	Yes	Yes
**Dependent variables:** Ln (Support_c-p_)				
URRBMI	0.678*** (0.086)	0.016 (0.144)	0.093 (0.109)	0.253*** (0.087)
Observations	43,611	11,451	26,361	28,701
*R*-squared	0.204	0.244	0.166	0.125
Covariates	Yes	Yes	Yes	Yes
Province fixed effects	Yes	Yes	Yes	Yes
Year fixed effects	Yes	Yes	Yes	Yes

*, **, and *** indicate that the coefficients significantly differ from 0 at the 10%, 5%, and 1% levels. The dependent variables are scaled by it’s logarithmic form

The results in Columns (3) and (4) of Table 3 show that family financial support from parents to children increases significantly by 48.2% for elderly URRBMI enrollees, while this amount increases by 52.2% for middle-aged enrollees. What’s more, the effect of URRBMI on family financial support from children to parents is significant among elderly enrollees and nonsignificant among middle-aged enrollees.

### Robustness tests

In the first robustness test, we use three matching methods: nearest neighbor matching (k = 4), radius matching, and kernel matching. The balance check of the distribution of the covariates between the treatment group and control group is shown in Table 4. The matching strategy is effective since the results indicate that all the covariates in the post-matching subsample pass the balancing test since the absolute standardized bias of the covariates is less than 10% [41]. Table 5 reports the ATT of URRBMI. The PSM estimates based on three different matching methods show that the ATT of URRBMI on family financial support from parents to children is 0.641, and the ATT of URRBMI on family financial support from children to parents is 0.959. Our results remain robust while the OLS estimator underestimates the effect of URRBMI. The heterogeneous results show that URRBMI has a positive and significant effect on rural, urban, middle-aged, and elderly enrollees. However, this effect on rural enrollees’ financial support, middle-aged enrollees’ financial support to children, and the elderly’s financial support received from children is more remarkable.

**Table 4:** Covariates balancing test of PSM: Mean differences before and after matching

**Variable**	**Mean**				**% reduction**
		Treated	Control	% bias	|bias|
Age	U	60.664	61.509	-8.4	46.0
	M	60.664	61.12	-4.5	
Male	U	0.480	0.428	10.5	89.6
	M	0.480	0.475	1.1	
Urban resident	U	0.203	0.312	-25	95.5
	M	0.203	0.208	-1.1	
Marriage status	U	0.883	0.806	21.3	82.5
	M	0.883	0.869	3.7	
No formal education	U	0.245	0.302	-12.9	95.1
	M	0.244	0.242	0.6	
Can read and write	U	0.197	0.221	-6	96.9
	M	0.197	0.197	0.2	
Primary school	U	0.226	0.200	6.5	72
	M	0.226	0.219	1.8	
Secondary school or above	U	0.332	0.277	12	80
	M	0.332	0.343	-2.4	
Public pensions	U	0.602	0.294	65.1	98.5
	M	0.602	0.606	-1	
Self-rated health status	U	3.042	3.083	-4.2	46.8
	M	3.042	3.020	2.3	
Number of children	U	3.029	3.162	-7.7	74
	M	3.029	3.063	-2	
Living arrangement	U	0.280	0.349	-15	80.8
	M	0.279	0.293	-2.9	
Family financial assets	U	0.047	0.031	6.6	89.2
	M	0.047	0.045	0.7	

% bias denotes mean standardized difference in percentage. “U” represent “the unmatched subsample”, and “M” represent “the matched subsample”. The balancing test results here are from nearest neighbor matching (k = 4) of the whole sample. The matching test results of radius matching, kernel matching and other sub-groups are also effective, which are available upon request

**Table 5:** The ATT of URRBMI on family financial support: PSM estimates

**Dependent variables**	**Nearest neighbor matching (k = 4)**		**Radius matching**		**Kernel matching**		The average of ATT
	ATT	t-statistic	ATT	t-statistic	ATT	t-statistic	
Ln (Support_p-c_)							
Full sample	0.639***	6.04	0.685***	7.21	0.600***	7.23	0.641
Rural	0.719***	3.61	0.793***	4.39	1.019***	6.27	0.844
Urban	0.664***	5.62	0.626***	5.82	0.588***	6.26	0.626
Middle-aged	0.753***	4.06	0.697***	4.19	0.610***	4.33	0.687
Elderly	0.496***	4.06	0.556***	5.10	0.552***	5.63	0.535
Ln (Support_c-p_)							
Full sample	0.879***	7.53	0.943***	8.99	1.056***	11.55	0.959
Rural	0.974***	7.13	0.922***	7.40	1.018***	9.38	0.971
Urban	0.728***	3.60	0.786***	4.29	0.893***	5.42	0.802
Middle-aged	0.681***	4.66	0.838***	6.43	0.957***	8.18	0.825
Elderly	1.128***	6.16	1.149***	6.98	1.184***	8.50	1.154

*, **, and *** indicate that the coefficients significantly differ from 0 at the 10%, 5%, and 1% levels, respectively. The dependent variables are scaled by it’s logarithmic form. ATT means the Average Treatment Effect on the Treated

In the second robustness test, we use ${URRBMI}_{coverage}$ as the independent variable to rule out reverse causality. The results in Table 6 report the effect of ${URRBMI}_{coverage}$ on family financial support and the results remain robust when compared with the results from Tables 2 and 3.

**Table 6:** The robustness test: the effects of URRBMI Coverage on family financial support

	**Ln (Support**_**p-c**_**)**	**Ln (Support**_**p-c**_**)**	**Ln (Support**_**c-p**_**)**	**Ln (Support**_**c-p**_**)**
	(1)	(2)	(3)	(4)
URRBMI coverage	0.993*** (0.219)	0.680*** (0.200)	0.674*** (0.233)	0.535** (0.226)
Observations	15,349	15,349	15,349	15,349
*R*-squared	0.022	0.187	0.022	0.097
Covariates	No	Yes	No	Yes
Province fixed effects	Yes	Yes	Yes	Yes
Year fixed effects	Yes	Yes	Yes	Yes
	**Urban and rural residence**		**Age**	
	**Rural**	**Urban**	**Middle-aged**	**Elderly**
**Dependent variables:** Ln (Support_p-c_)				
URRBMI coverage	0.535** (0.257)	0.220 (0.477)	0.758*** (0.276)	0.119 (0.361)
Observations	12,181	3,168	6,542	8,807
*R*-squared	0.091	0.102	0.053	0.108
Covariates	Yes	Yes	Yes	Yes
Province fixed effects	Yes	Yes	Yes	Yes
Year fixed effects	Yes	Yes	Yes	Yes
**Dependent variables:** Ln (Support_c-p_)				
URRBMI coverage	0.865*** (0.230)	0.248 (0.407)	0.297 (0.314)	0.896*** (0.255)
Observations	12,181	3,168	6,542	8,807
*R*-squared	0.165	0.250	0.192	0.125
Covariates	Yes	Yes	Yes	Yes
Province fixed effects	Yes	Yes	Yes	Yes
Year fixed effects	Yes	Yes	Yes	Yes

*, **, and *** indicate that the coefficients significantly differ from 0 at the 10%, 5%, and 1% levels. The dependent variables are scaled by it’s logarithmic form

### The mediation analysis

In the previous section, we find that enrollment in URRBMI has a significant effect on family financial support between the insured parents and their children. We examine the indirect effects of URRBMI through the out-of-pocket ratio of medical expenses and health expense in this section. First, enrollment in URRBMI could reduce the out-of-pocket ratio of medical expenses and alleviate the burden of medical expenses. As a result, parents who are enrolled in URRBMI can provide more financial support to their children. Second, URRBMI may raise individuals’ health awareness and stimulate health consumption, thus crowding in children’s financial support to parents.

The results in Columns (1) to (3) of Table 7 report the regression results of the out-of-pocket ratio of inpatient medical expenses as a mediator. Column (1) shows the effect of URRBMI on the out-of-pocket ratio of inpatient medical expenses. The estimated coefficient on URRBMI is −0.03, which indicates that the out-of-pocket ratio decreases significantly if the respondent is enrolled in URRBMI. Column (2) shows the total effect of URRBMI on family financial support from parents to children. As explained above, the total amount of family financial support increases by 18.7% after enrolling in URRBMI. The last step of the mediation analysis is to test the impact of URRBMI on family financial support by including the out-of-pocket ratio as a covariate. The results in Column (3) show that after controlling for the impact of the out-of-pocket ratio, the effect of URRBMI on family financial support is significant. However, it drops from 0.187, the result in Column (2), to 0.177. Therefore, we conclude that URRBMI has a crowd-in effect on family financial support from parents to children, and part of this crowd-in effect is mediated by a reduction in the out-of-pocket ratio of inpatient medical expenses.

**Table 7:** The mediation analysis of URRBMI on family financial support

	**Medical expenses**_**Out-of-pocket ratio**_	**Ln (Support**_**p–c**_**)**	**Ln (Support**_**p–c**_**)**	**Ln (Health expenses)**	**Ln (Support**_**c-p**_**)**	**Ln (Support**_**c-p**_**)**
	(1)	(2)	(3)	(4)	(5)	(6)
URRBMI	-0.030*** (0.003)	0.187*** (0.070)	0.177** (0.070)	0.306*** (0.053)	0.518*** (0.074)	0.504*** (0.074)
Medical expenses_Out-of-pocket ratio_			-0.335*** (0.095)			
Ln (Health expenses)						0.048*** (0.006)
Observations	55,062	55,062	55,062	55,062	55,062	55,062
*R*-squared	0.053	0.151	0.151	0.103	0.216	0.217
Covariates	Yes	Yes	Yes	Yes	Yes	Yes
Province fixed effects	Yes	Yes	Yes	Yes	Yes	Yes
Year fixed effects	Yes	Yes	Yes	Yes	Yes	Yes

*, **, and *** indicate that the coefficients significantly differ from 0 at the 10%, 5%, and 1% levels. The dependent variable is scaled by it’s logarithmic form. Family financial assets are scaled by 1/100,000

Next, we examine whether URRBMI has a crowd-in effect on family financial support from children to parents by stimulating health consumption. The results presented in Columns (4) to (6) of Table 7 indicate that health consumption increases by 30.6% after enrolling in URRBMI, which corroborates earlier findings that medical insurance has a positive effect on consumption related to health investment and health care. The results in Column (6) show that after controlling for the impact of health consumption, the effect of URRBMI on family financial support is significant, and its coefficient decreases from 0.518 to 0.504. Therefore, URRBMI has a crowd-in effect on family financial support from children to parents by increasing the health consumption of insured parents.

The results of the Sobel-Goodman mediation tests are given in Table 8. The results shown in the upper part of Table 8 suggest that the out-of-pocket ratio of inpatient medical expenses is an effective mediator. The negative coefficient *a* indicates that enrollment in URRBMI significantly reduces the out-of-pocket ratio of inpatient medical expenses by 3.0%, which is consistent with the results in Table 7. The negative coefficient *b* indicates that a 10% decrease in the out-of-pocket ratio significantly increases family financial support from parents to children by 3.35%. Therefore, URRBMI promotes family financial support from parents to children by reducing the out-of-pocket ratio of inpatient medical expenses. This positive mediating role accounts for 5.4% of the total effect. The results of health consumption shown in the second part of Table 8 indicate that URRBMI has a positive effect on health consumption and the increase of health consumption could further increase financial support from children to parents. In addition, the indirect effect, i.e., the mediation effect of health consumption, is statistically significant, with approximately 2.8% of the total effect being mediated.

**Table 8:** The Sobel-Goodman Mediation Tests of URRBMI on family financial support

Mediator: **Medical expenses**_**Out-of-pocket ratio**_	Coefficient	Std Err	Z	*P* > Z
a coefficient	-0.030	0.004	-8.452	0.000
b coefficient	-0.335	0.091	-3.680	0.000
Indirect effect	0.010	0.003	3.374	0.001
Direct effect	0.177	0.076	2.319	0.020
Total effect	0.187	0.076	2.453	0.014
Proportion of total effect that is mediated: 0.054				
Ratio of indirect to direct effect: 0.057				
Ratio of total to direct effect: 1.057				
Mediator: **Ln (Health expenses)**	Coefficient	Std Err	Z	*P* > Z
a coefficient	0.306	0.053	5.725	0.007
b coefficient	0.048	0.006	8.292	0.000
Indirect effect	0.015	0.003	4.711	0.000
Direct effect	0.504	0.073	6.955	0.000
Total effect	0.519	0.073	7.155	0.000
Proportion of total effect that is mediated: 0.028				
Ratio of indirect to direct effect: 0.029				
Ratio of total to direct effect: 1.029				

### The moderation analysis

The results in Table 9 indicate the coefficient of the interaction term of education and URRBMI is significantly positive, which means the education of parents has a moderating effect on the relationship between URRBMI and family financial support from parents to children. A high level of parental education is conducive to increasing the financial support from parents to children in the insured group. The health status of parents also has a moderating effect on the relationship between URRBMI and family financial support from children to parents. Insured parents with a better health status receive significantly less financial support from their children than those whose health is worse.

**Table 9:** The hierarchical multiple regression results for the moderation analysis

	**Ln (Support**_**p-c**_**)**	**Ln (Support**_**p-c**_**)**	**Ln (Support**_**p-c**_**)**	**Ln (Support**_**c-p**_**)**	**Ln (Support**_**c-p**_**)**	**Ln (Support**_**c-p**_**)**
	(1)	(2)	(3)	(4)	(5)	(6)
URRBMI	0.229*** (0.070)	0.187*** (0.070)	-0.017 (0.220)	0.524*** (0.074)	0.518*** (0.074)	0.972*** (0.252)
Education		0.221*** (0.016)	0.091 (0.059)		0.035** (0.015)	0.042 (0.062)
Self-rated health status		0.127*** (0.016)	0.126* (0.067)		0.032** (0.015)	0.169** (0.074)
URRBMI*Education			0.136** (0.059)			-0.007 (0.062)
URRBM*Self-rated health status			0.002 (0.069)			-0.144* (0.075)
Observations	55,062	55,062	55,062	55,062	55,062	55,062
*R*-squared	0.146	0.150	0.151	0.216	0.216	0.216
Covariates	Yes	Yes	Yes	Yes	Yes	Yes
Province fixed effects	Yes	Yes	Yes	Yes	Yes	Yes
Year fixed effects	Yes	Yes	Yes	Yes	Yes	Yes

*, **, and *** indicate that the coefficients significantly differ from 0 at the 10%, 5%, and 1% levels. The dependent variable is scaled by it’s logarithmic form. Family financial assets are scaled by 1/100,000

## Discussion

The empirical findings in this paper make several contributions to the understanding of public program, family decisions, and family welfare. Before this study, empirical evidence on the positive effect of medical insurance on intergenerational support within Chinese family was limited. Our study is one of the first attempts to thoroughly examine the impact of medical insurance on family financial support. Through our evidence, we confirm the positive effect of URRBMI on personal financial status and family welfare. Our results indicate that URRBMI could significantly reduce the out-of-pocket ratio of inpatient medical expenses, thus reducing the burden of medical expenses. We also demonstrate that URRBMI has a positive effect on increasing the health investment and health consumption, which may further improve people’s health. Different from the previous literature, which mainly focuses on the preliminary effect of URRBMI on health and personal finance [1, 2, 7, 9, 23], we further examine the positive effect of URRBMI on intergenerational financial support within the family. Since the family financial support between parents and children is an important symbol of family happiness and harmony in China, we believe that the positive effect of medical insurance on family welfare may be underestimated in previous studies. Our analysis also contributes to the literature on the evaluation of the spillover effect of medical insurance policies on non-target populations, such as the study of the indirect effect of medical insurance on increasing labor supply and releasing physical stress for the family members of the insured [42].

In addition, the results from the heterogeneity effect are also striking. The positive effect of URRBMI on family financial support is more significant among rural residents. This may be related to the fact that rural residents in China place more importance on family solidarity and support. The previous study has indicates that the core functions of the family as the major welfare provider in rural China have remained [43]. Therefore, after enrolling in URRBMI, rural parents are more willing to increase financial and material assistance to their families. This finding is also consistent with the study of attitudes toward family obligation among urban and rural adolescents, which has shown that urban male adolescents have a weaker sense of family obligation than do rural male adolescents [44]. Fueled by a stronger sense of family obligation, children in rural area also increase more financial support to their parents. Besides, the positive effect of URRBMI is also more significant in middle-aged parents’ financial support to their children, and children’s financial support to their elderly parents. And this heterogeneity by age can be explained by the difference in health and economic status of people at different ages. Generally, younger parents tend to have better physical and economic conditions than the elderly. Thus, after the enrollment in URRBMI, middle-aged parents have an advantage over elderly ones to provide more financial support to their children.

It is important to note some limitations in our study. First, although one of the strengths of our data is that CHARLS data are nationally representative, this data may not objectively reflect some characteristics of respondents. Slight errors may have occurred when a respondent was asked to recall the money and in-kind support between his/her children. In addition, the use of self-rated health status as a representation of physical health status may bring some deviation. Second, we consider that medical insurance has a positive effect on family harmony since it can increase financial support between family members. However, we did not measure this welfare improvement effect by an individual or family utility function. Thus, there are some difficulties in comparing the effects of different medical insurance policies. Future studies might introduce an expected utility model and measure the utility improvement of this effect.

## Conclusion

This article uses data from CHARLS for the years 2011–2018 to estimate the effect of URRBMI on family financial support. The results show that URRBMI significantly improves financial support between insured parents and their children. The out-of-pocket ratio of inpatient medical expenses and the health expense of insured parents are important mechanisms that mediate the effect of URRBMI on family financial support. In addition, both the heterogeneity effect and moderation effect are discussed in our study, providing a comprehensive understanding of the effect of URRBMI on different family backgrounds. Overall, URRBMI could significantly promote financial support between family members, which we believe is conducive to family harmony and intergenerational mutual assistance.

The results from our study have several implications for the literature on medical insurance and other social welfare programs, and for literature focused on family economics and labor economics. Our results suggest that it is important to recognize the roles of both preliminary and spillover results in the evaluation of public policy. The medical program may not only influence the benefits of beneficiaries, but also affect their cognition and decision-making through a direct or mediation mechanism. In addition, the government and policymakers should pay attention to the fact that the benefits of a public program can be moderated by other factors. Therefore, it is important to consider the needs of different groups when developing and evaluating public policies.

## Data Availability

The datasets generated and analysed during the current study are available in the CHARLS repository, (http://charls.pku.edu.cn/en).

## Abbreviations

CHARLS: China Health and Retirement Longitudinal Study
URRBMI: Urban and Rural Resident Basic Medical Insurance
CII: Critical Illness Insurance
NCMS: New Rural Cooperative Medical Scheme
UEMI: Urban Employee Medical Insurance
URMI: Urban Resident Medical Insurance

## Footnotes

1 Data Sources: Statistics from the National Healthcare Security Administration. (http://www.nhsa.gov.cn)
